# Separation of Alcohol-Water Mixtures by a Combination of Distillation, Hydrophilic and Organophilic Pervaporation Processes

**DOI:** 10.3390/membranes10110345

**Published:** 2020-11-16

**Authors:** Huyen Trang Do Thi, Peter Mizsey, Andras Jozsef Toth

**Affiliations:** 1 Environmental and Process Engineering Research Group, Department of Chemical and Environmental Process Engineering, Budapest University of Technology and Economics, Műegyetem rkp. 3, H-1111 Budapest, Hungary; dothihuyentrang@edu.bme.hu (H.T.D.T.); mizsey.miskolc@gmail.com (P.M.); 2 Institute of Chemistry, University of Miskolc, Egyetemváros C/1 108, H-3515 Miskolc, Hungary

**Keywords:** process wastewater, ethanol-water separation, methanol-water separation, pervaporation, hybrid operation

## Abstract

It can be stated that in the fine chemical industries, especially in the pharmaceutical industry, large amounts of liquid waste and industrial waste solvents are generated during the production technology. Addressing these is a key issue because their disposal often accounts for the largest proportion of the cost of the entire technology. There is need to develop regeneration processes that are financially beneficial to the plant and, if possible, reuse the liquid waste in the spirit of a circular economy, in a particular technology, or possibly elsewhere. The distillation technique proves to be a good solution in many cases, but in the case of mixtures with high water content and few volatile components, this process is often not cost-effective due to its high steam consumption, and in the case of azeotropic mixtures there are separation constraints. In the present work, the membrane process considered as an alternative; pervaporation is demonstrated through the treatment of low alcohol (methanol and ethanol) aqueous mixtures. Alcohol-containing process wastewaters were investigated in professional process simulator environment with user-added pervaporation modules. Eight different methods were built up in ChemCAD flowsheet simulator: organophilic pervaporation (OPV), hydrophilic pervaporation (HPV), hydrophilic pervaporation with recirculation (R-HPV), dynamic organophilic pervaporation (Dyn-OPV), dynamic hydronophilic pervaporation (Dyn-HPV), hybrid distillation-organophilic pervaporation (D + OPV), hybrid distillation-hydrophilic pervaporation (D + HPV), and finally hybrid distillation-hydrophilic pervaporation with recirculation (R-D + HPV). It can be stated the last solution in line was the most suitable in the terms of composition, however distillation of mixture with high water content has significant heat consumption. Furthermore, the pervaporation supplemented with dynamic tanks is not favourable due to the high recirculation rate in the case of tested mixtures and compositions.

## 1. Introduction

Nowadays, one of the most important problems is the protection of the quality and quantity of our water resources. Unlimited amounts of water have been available since man’s appearance. On the other hand, water demand is increasing day by day, as the population, cultural and social needs are also increasing, as well as the rapid industrial development that is taking place. Pollution of natural waters is mainly caused by industrial plants and agricultural activities. Industrial wastewater is causing increasing difficulties, which is why regulations for wastewater treatment are also becoming more stringent to protect the environment. These rules force emitters to reduce emissions of various industrial pollutants, to recycle, and use valuable by-products and waste using new technology. Separation of various organic substances used in industry, such as alcohol, from process wastewater is an important task of environmental protection.

Separation of liquid waste mixtures is a field that has been significantly and thoroughly studied not only for environmental engineering but also for other engineering sciences. Liquid mixtures are often non-ideal, with very different behaviour from the ideal. In many cases, they form an azeotrope that cannot be separated by conventional distillation methods. Separation of non-ideal azeotrope-containing mixtures is often complex and serious challenge. Therefore, there is need for hybrid process that can efficiently and economically separate azeotropic mixtures, such as pressure change, extractive, homogeneous azeotropic and heterogeneous azeotropic distillation [1,2,3,4,5,6,7,8,9], hybrid distillation-pervaporation process [10,11,12,13,14,15] extractive heterogeneous azeotropic distillation (EHAD) [16,17,18] and hydrophilic or organophilic pervaporation (HPV, OPV) [19,20,21,22,23,24]. It can be mentioned that volatile organic compounds (VOCs) [25,26] can be separated from wastewater by pervaporation membranes and distillation processes, e.g., ethyl acetate-ethanol [27,28], acetone-butanol-ethanol [29], isobutanol [30,31], isopropanol [32,33,34,35], tetrahydrofuran (THF) [36], ethanol [37,38,39], methanol [35,40].

The hybrid distillation-pervaporation process is considered as a clean technology and it has potential savings in energy because of reduced thermal and pressure requirements. This process allows using the heat of the distillation to increase the efficiency of the pervaporation process and leads consequently to potential savings in energy costs [41]. So, this hybrid separation process is energetically more efficient compared to conventional distillation.

Tusel and Ballweg [42] examined a system combining distillation column followed by two pervaporation units with different types of hydrophilic membrane. The first step was a ‘high flux-low selectivity’ membrane to split the azeotrope mixtures. The second step was a ‘low flux-high selectivity’ membrane as a polishing. In this separation process, the pervaporation membrane modules were operated at 72 °C, 3 bars. The feed at 15 °C contained 8.8% by weight ethanol and 12,720 kg/h. Ethanol was concentrated from 8.8 w% to 99.8 w%. The ethanol flow was 1103 kg/h. In addition to the hybrid distillation-pervaporation process, pervaporation can also be combined with other systems to separate mixtures, e.g., pervaporation-crystallization (PC) process [43], pervaporation-microfiltration-osmotic distillation three-stage hybrid process [44], reverse osmosis-pervaporation hybrid process [45].

Pervaporation is a membrane operation where a phase change occurs. In the last ten years, pervaporation has been considered one of the most dynamically developing membrane separation operations. The main advantage of pervaporation is the energy-saving operation. The pervaporation requires lower energy consumption than other technologies, in many cases 50–70% less [46]. Further, this is an environmentally friendly operation because not require to use of additional chemicals or materials [47]. The pervaporation membrane can be used to separate azeotropic mixtures. Nowadays, within the more stringent requirements of sustainable development, the environmentally friendly technology of pervaporation can provide a concrete response and a real solution for many separation processes, even on a larger, industrial scale [48,49,50,51]. The pervaporation process is used to dehydrate organic compounds [52,53,54,55,56], to remove small amounts of undesirable organic compounds from water-organic mixture [56,57,58,59], and to separate organic compounds from an organic mixture [60,61,62,63]. The water-alcohol separation was first used to study and apply the pervaporation process in the chemical industry [64,65]. The main future trends can be structured in two research strategies [66]:

Approach I.: Improving the predictive power of mass flow models in pervaporation to extrapolate its operational performance under other conditions. These models can be implemented in the general simulation and optimization phase of hybrid processes that integrate pervaporation with other separation units (pervaporation-distillation).

Approach II.: Simulation and optimization of hybrid processes, calculation of the required membrane performance. Empirical or semiempirical simple models can then be used under the selected operating conditions to obtain the information needed to achieve the required membrane performance (effect of temperature, material, microstructure, etc.).

**Figure 1:**
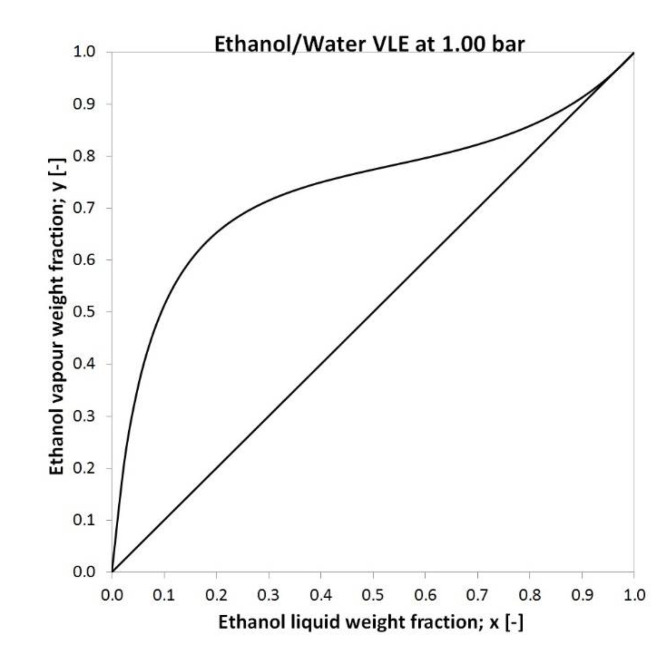
Ethanol-water mixture vapour-liquid equilibrium diagram at 1 bar [67].

The ethanol-water mixture can be considered as a minimum boiling point homogeneous azeotropic. Figure 1 shows the equilibrium diagram of the ethanol-water mixture. The azeotropic composition depends on the pressure. By changing the pressure, the azeotropic nature of the system may cease. The ethanol-water mixture has a so-called ethanol content of 95.63 w% azeotropic point at 1 bar [48].

Pervaporation of ethanol-water mixtures using hydrophilic zeolite NaA membranes was reported by Shah et al. [68]. The total flux for the ethanol-water mixture was found to vary from 2 to 0.05 kg/m^2^/h at 60 °C as the feed solvent concentration was increased from 0 to 100 w%. The zeolite membranes exhibit high selectivities (separation factors between 1000 and 5000) over the entire range of ethanol concentrations [68]. Pervaporation separation of alcohol-water mixtures includes ethanol-water with PDMS/PTEE membrane has also been studied by Zhang et al. [69]. The PDMS/PTEE membrane was made of polydimethylsiloxane (PDMS) cross-linked with *n*-heptane on a polytetrafluoroethylene (PTFE) membrane substrate with a thickness of approximately 50 μm. The feed at 30 °C was 2 w% ethanol with a separation factor value of 10.

**Figure 2:**
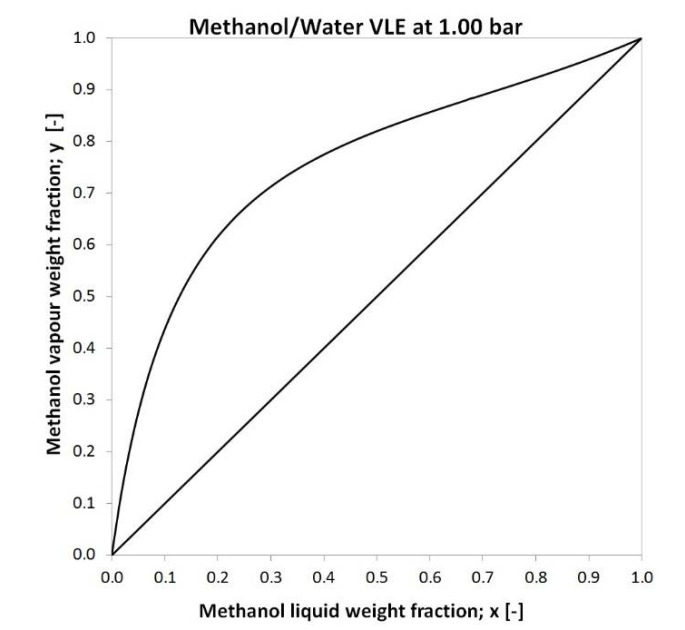
Methanol-water mixture vapour-liquid equilibrium diagram at 1 bar [70].

The methanol-water mixture is considered a near-ideal zeotropic mixture, typical mixtures of homologues. The most important property of an ideal solution is additivity: mass, volume, and components can be calculated by simple summation. It does not change during the formation of the mixture, and the properties of the finished mixture can be calculated by simple summation, taking into account the mixing ratio. The ideal and near-ideal mixture is more easily separated than the azeotropic mixture. Figure 2 shows the vapour-liquid equilibrium diagram of methanol-water mixture.

Liu et al. have studied the membrane pervaporation of water from a methanol-water mixture with a polyvinyl alcohol (PVA) and nanometer SiO_2_ membrane [71]. In their study, PVA/SiO_2_ membranes were used to separate mixtures of methanol-water over the complete concentration range of 70–98%. For the 98% mixture at 60 °C, the separation factor is up to 1458 together with a permeate flux up to 325 g/(m^2^·h). The evaluation of PDMS (PERVAP-1060) membrane to separate methanol from aqueous solutions has been performed by Kujawski [72]. PERVAP-1060 is one of the organophilic membranes, which also showed prospective potential in selective and transport properties. In his work, the operating temperature was set at 30 °C together with the feed methanol concentration of 5 w%. The calculated permeate methanol concentration was 24 w% with a separation factor of 5.

Pervaporation and hybrid distillation-pervaporation process are widely regarded as an attractive and efficient technology for various separation processes, therefore several combinations were examined to select the most efficient. The aim of this work is to model the hybrid distillation-pervaporation, hydrophilic and organophilic pervaporation processes in the ChemCAD professional flowsheet simulator. The main novelty of research is the investigation of pervaporation in a dynamic model environment too. The UNIQUAC thermodynamic model was used for modelling distillation processes. The separation of the binary alcohol-water mixtures was studied, the near-ideal methanol-water mixture and the homogeneous azeotropic ethanol-water mixture with minimum boiling point. This study aimed to compare the separation methods with the collected data, taking into account different aspects, indicating the obtained values.

## 2. Materials and Methods

The aim of the alcohol-water (ethanol-water and methanol-water) separation of the given composition was to achieve the purest possible products. Eight different methods were investigated in ChemCAD flowsheet simulator, which is listed in Table 1.

### 2.1. Modelling Schemes

**Figure 3:**
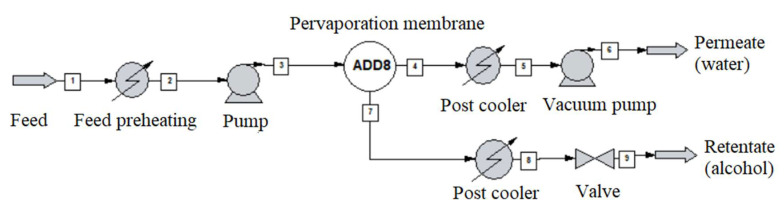
Flowsheet of hydrophilic pervaporation membrane (in case of organophilic reverse product available).

**Figure 4:**
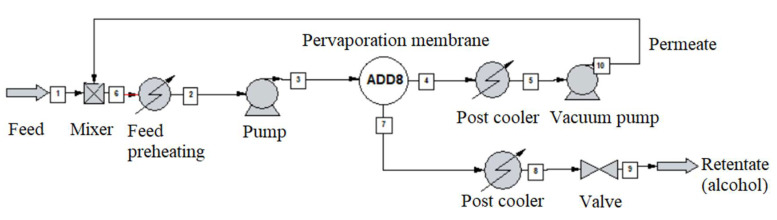
Flowsheet of the recirculating hydrophilic pervaporation method.

**Figure 5:**
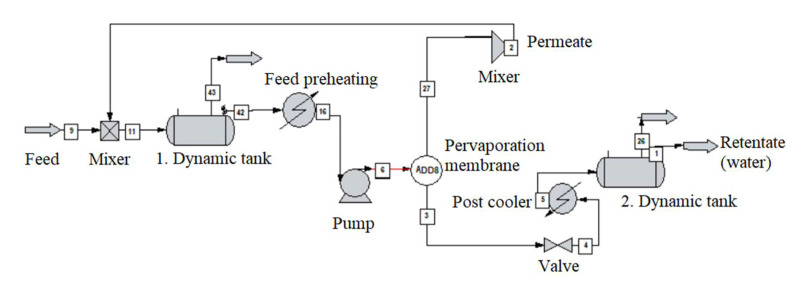
Flowsheet of dynamic organophilic pervaporation method.

**Figure 6:**
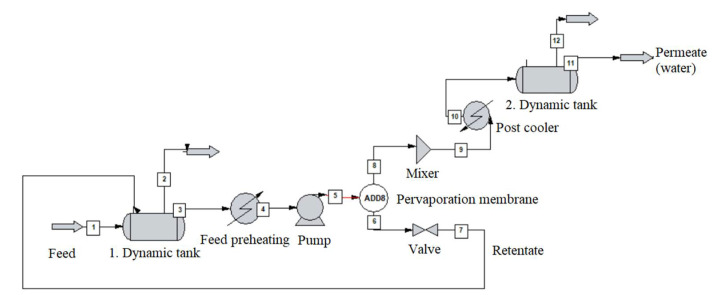
Flowsheet of dynamic hydrophilic pervaporation method.

**Figure 7:**
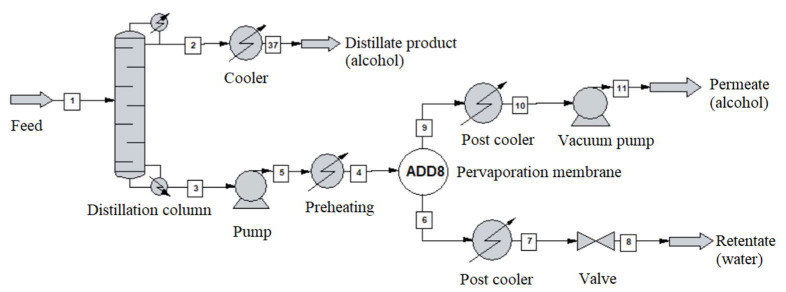
Flowsheet of hybrid distillation-organophilic pervaporation method.

**Figure 8:**
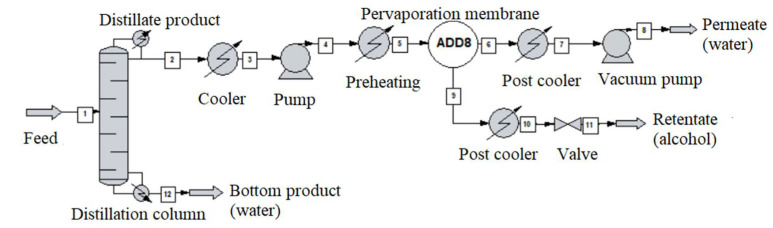
Flowsheet of hybrid distillation-hydrophilic pervaporation method.

**Figure 9:**
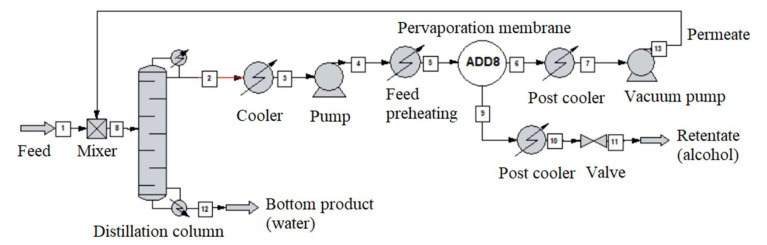
Flowsheet of the recirculation hybrid distillation-hydrophilic pervaporation method.

The alcohol-water mixture separation methods are shown in Figure 3, Figure 4, Figure 5, Figure 6, Figure 7, Figure 8 and Figure 9. The hydrophilic pervaporation membrane procedure is presented in Figure 3, the recirculating hydrophilic pervaporation procedure is presented in Figure 4. In Figure 5 and Figure 6, the dynamic organophilic pervaporation method and dynamic hydrophilic pervaporation method are shown respectively. The hybrid distillation-organophilic pervaporation method and hybrid distillation-hydrophilic pervaporation method is shown in Figure 7 and Figure 8. And finally, the recirculation hybrid distillation-hydrophilic pervaporation method is presented in Figure 9. From hydrophilic systems, water can be obtained in principle as a permeate product and ethanol as a retentate product. The recirculation cases were also examined.

### 2.2. Membrane Characteristics, Feed Data

In this paper, the properties of pervaporation membrane are adopted from the experiments performed by the Environmental and Process Engineering Research Group of BME, shown in Table 1, Table 2, Table 3 and Table 4. Equation (1) shows the equation of developed Rautenbach model [73,74] on which the pervaporation calculation is based:

(1) $$J_{i}=\frac{1}{1+\left\{\frac{\left[\;{\bar{D}}_{i}\cdot \exp\left(B\cdot x_{i1}\right)\right]}{\left(p_{i0}\cdot {\bar{\gamma }}_{i}\right)}\right\}}\cdot \frac{\left[{\bar{D}}_{i}\cdot \exp\left(B\cdot x_{i1}\right)\right]}{{\bar{\gamma }}_{i}}\cdot \left(\frac{p_{i1}-p_{i3}}{p_{i0}}\right)\;i=\left(1,\ldots ,k\right)$$

Three different polymer membranes were experimentally examined for application of membrane flowsheet models. PERVAP™ 1210 (Table 2) and PERVAP™ 1510 (Table 4) are hydrophilic pervaporation membranes to separate ethanol-water or methanol-water mixtures. PERVAP™ 4060 (Table 3, Table 4 and Table 5) is an organophilic pervaporation membrane used to separate the alcohol-water mixtures. The experimental results have published in [70,74,75,76]. Table 2, Table 3, Table 4 and Table 5 summarize the optimized parameters of experimental investigations. These parameters were used to build up the semi-empirical model (see Equation (1)) in the ChemCAD flowsheet program. The other major pervaporation models in the literature is the following: solution–diffusion model, total solvent volume fraction model and poreflow model [74].

Table 6 and Table 7 show the distillation column and dynamic tank parameters. In the Table 8, feed parameters are also shown. The feed at 20 °C, 1 bar contains 0.02 m/m alcohol (ethanol or methanol) and 0.98 m/m water.

### 2.3. Pervaporation System

In the first step, the optimization of the pervaporation membrane was performed (according to Figure 3). First of all, the type of pervaporation membrane was decided: hydrophilic pervaporation method (HPV) or organophilic pervaporation (OPV). In these analyses, the same input parameters were applied, a mixture of given mass flow and composition, 1000 kg/h liquid flow, water-alcohol in 0.98 and 0.02 m/m (see Table 8). The effective size of membrane area was set as changing variable. In each module, the surface area was 40 m^2^ of the pervaporation membrane. The feed flow into each membrane unit was kept at 70 °C by using heat exchanger. The feed pressure was kept at 3 bar using pump. The recirculation case was also investigated (see Figure 6).

### 2.4. Hybrid Distillation-Pervaporation System

In the next step, hybrid distillation-pervaporation system was simulated, see Figure 4. It is similar to the previous model, the first step was choosing the type of pervaporation membrane. A standard size column with 10 theoretical plates was modelled and the mixture was pumped into the fifth plate. The 1000 kg/h input of the corresponding alcohol-water composition, see Table 8, was maintained. The recirculation case was also performed (see Figure 7).

### 2.5. Dynamic Pervaporation System

The flowsheet of dynamic pervaporation system can be seen in Figure 5. Table 7 summarizes the parameters of dynamic tank. Vertical, flat-bottomed tank was used with the following dimensions, 5 m diameter, 10 m height, the liquid in the tank was 2 m high. The liquid in the tank was passed through a pervaporation membrane apparatus with a uniform flow of 1000 kg/h of the appropriate composition after the pressure has been increased to 3 bar by pumping and the liquid is heated to 70 °C and its operation was adiabatic. The vapour was separated on the stages (at 0.008 bar pressure) and the product was combined, compressed, condensed, and collected in another tank. The liquid exiting of pervaporation was expanded and recycled into the starter at the pressure of feed condition. The simulation time was set at 10 h with 1-min increments each step.

## 3. Results and Discussion

**Figure 10:**
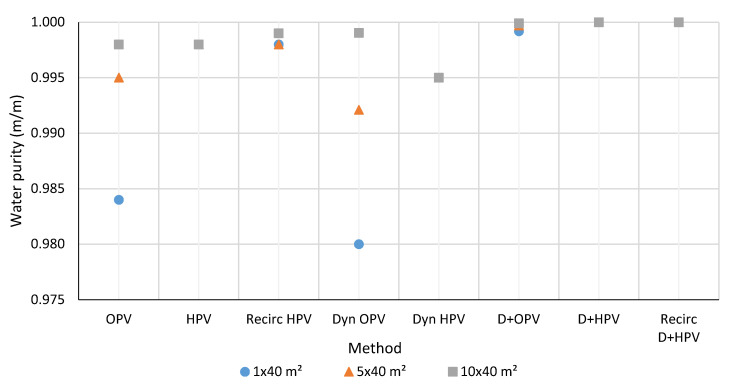
Comparison of the water purity of ethanol-water selection methods.

**Figure 11:**
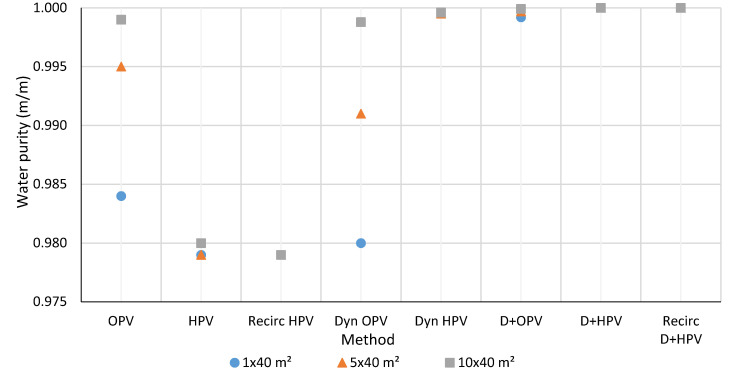
Comparison of the water purity of methanol-water separation methods.

**Figure 12:**
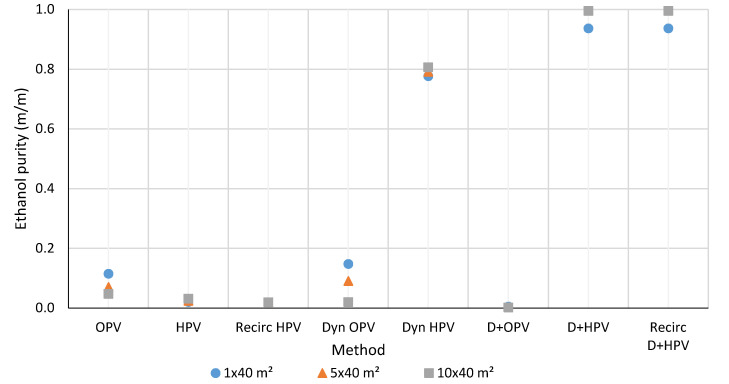
Comparison of the ethanol purity of ethanol-water purification methods.

**Figure 13:**
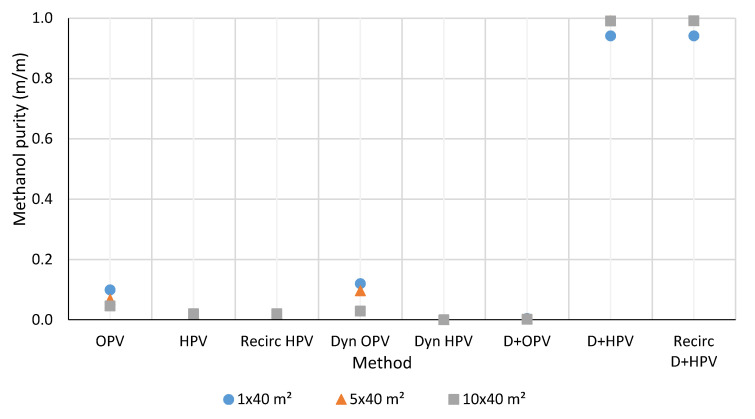
Comparison of the methanol purity of methanol-water purification methods.

The detailed results and data of each investigated model are presented in the Supplementary Part. The results of three systems, 1 × 40 m^2^, 5 × 40 m^2^, and 10 × 40 m^2^ effective membrane area are shown in Figure 10, Figure 11, Figure 12 and Figure 13. The graphs are with a standard error of ± 0.05%. Water is obtained from hydrophilic systems as permeate product and ethanol or methanol as retentate product. In the case of organophilic systems, products are obtained in reverse order. It is expected to give higher purity of ethanol and water using a hydrophilic membrane than an organophilic one, due to the higher separation efficiency.

### 3.1. Water Purity

The available water purity in the case of ethanol-water mixture is shown in the Figure 10 and the results of methanol-water binary mixture can be seen in Figure 11. Inferring from ethanol-water mixture selection systems, it can be said in general that the higher the number of pervaporation membranes in the system, the better the quality of water composition can be reached. The D+HPV and D+HPV recirculation methods provide the purest water (maximum achievable purity: 0.99999 m/m water). With the D+HPV method, ethanol-water separation is the most efficient way to separate water, followed by the D+HPV recirculation method, followed by the HPV, dynamic HPV method, and much worse with the organophilic membrane. In the case of the hydrophilic membrane, the water component is better separated on the permeate side.

In the methanol-water case, the D+HPV and D+HPV with recirculation methods provide even better water purity results. It can be observed, the more the number of pervaporation membranes that are in the system, the water composition quality is better. However, the HPV method simulation results of the methanol-water mixture show poorer water purity than in the case of the ethanol-water mixture. This observation can be explained by the fact that pervaporation is mainly used for the separation of azeotropic mixtures where a small amount of undesirable component has to be removed from the liquid mixture. Methanol dehydration process has a significantly lower separation factor value than ethanol dehydration, therefore distillation is a better recommended solution for methanol purification.

### 3.2. Ethanol and Methanol Purity

The available alcohol product purities of the three different systems are summarized in Figure 12 and Figure 13. As it can be seen, in the case of very dilute aqueous solutions, pervaporation alone is not suitable for enriching the alcohol content. The D+HPV and D+HPV recirculation methods provide good quality of both water and alcohol. After the distillation process, min. 90 w% of ethanol is obtained as the distillate product and 99.999 w% aqueous mixture as the bottom product. In the case of hydrophilic pervaporation membrane, the distillate product has further flowed through the pervaporation membranes. Hence, the better purity of alcohol is obtained compared with any other methods.

If we compare the results from the D+HPV or D+HPV recirculation systems with the results of Tusel and Ballweg [42], it can be seen that the ethanol quality increases from 2% to 99.6% (while the obtained product of Tusel et al. was 99.8% ethanol). However, the amount of ethanol obtained is 979.936 kg/h from 1000 kg/h input flow (accounting for 98% of the input mass amount), whereas using the system of Tusel et al., the amount of ethanol obtained was 1103 kg/h from 12,720 kg/h input (accounting for 8.7% of the input mass amount). The quality of ethanol or methanol obtained from the D+HPV or D+HPV recirculation system is greater than 99%. Therefore, it can be said that, compared with the published results of Zhang et al. [69], Liu et al. [71] and Tusel and Ballweg [42], the results obtained from the D+HPV and D+HPV recirculation systems are better in the product purity aspect.

On the other hand, the product purity hybrid D+HPV’s are better than the pervaporation-crystallization (PC) process [43], pervaporation–microfiltration–osmotic distillation hybrid process (PV+MF+OD) [44], reverse osmosis pervaporation-hybrid process (RO+PV) [45]. In the case of PC simulation [43], the feed flow rate was set at 500 kg/h containing ethanol (75 w%)/water (24 w%)/sodium pyruvate (1 w%) ternary mixture. The feed temperature and pressure were kept at 55–60 °C and 1 bar respectively. The feed mass was 14 kg. The products were obtained: 2.35 kg, 100 w% water accounting for 16.8% of the input mass amount and 8.3 kg, 4.42 w% water.

### 3.3. Heat Consumptions

In this section, the heat consumption of each method is analysed and summarized. The results are collected in Table 9. The heat consumption is calculated from the heat exchanger in the pervaporation systems, dynamic tank, and the heat requirements of the distillation column. The heat requirements for separation of the ethanol-water mixture and methanol-water mixture is nearly equal e.g., for D+HPV method with one pervaporation membrane unit the heat consumption for separation of ethanol-water is 325.86 MJ/h while in case of the methanol-water mixture is 325.83 MJ/h, see Table 9. The positive sign in Table 9 means the heat is given to the system and inversely the negative value means the heat is provided by the system. The more the membrane surface area increases, the more the amount of heat consumption also increases. The total heat consumptions in the cases of D+HPV and D+HPV with recirculation processes are positive, while for the other methods they are negative. From the simulation results, it is clear that the heat consumption in pervaporation methods is the smallest one, follows by hybrid distillation and pervaporation systems, respectively. There is a huge difference between the heat consumption using dynamic system compared to the others. Since in the dynamic system, the circulating flow is indeed extremely big compared to the input flow, and in some cases, the total flow is almost from the circulating e.g., Dyn HPV method the circulating flow is more than 90% of the total flow. Examining these results, the effectiveness of the dynamic system is questionable.

## 4. Conclusions

In summary, hydrophilic pervaporation membranes are much better suited for the separation of methanol-water and ethanol-water than organophilic pervaporation membranes. In the case of the hydrophilic membranes, the hybrid distillation-hydrophilic pervaporation system is the best solution for separating ethanol, methanol, and water, followed by a pervaporation process and a dynamic pervaporation process, respectively. In this present work, the pervaporation method with dynamic feed and product tanks were investigated in ChemCAD flowsheet environment for the first time. It can be stated that this solution is also capable of separating binary alcohol-water mixtures, however further investigation is needed to reduce the heat consumption and improve the recycle rate.

## Figures and Tables

**Table 1:** Studied methods.

Method	Abbreviation
Organophilic pervaporation	OPV
Hydrophilic pervaporation	HPV
Hydrophilic pervaporation with recirculation	Recirc HPV
Dynamic organophilic pervaporation	Dyn OPV
Dynamic hydronophilic pervaporation	Dyn HPV
Hybrid distillation-organophilic pervaporation	D + OPV
Hybrid distillation-hydrophilic pervaporation	D + HPV
Hybrid distillation-hydrophilic pervaporation with recirculation	Recirc D + HPV

**Table 2:** Hydrophilic pervaporation membrane parameters of ethanol-water mixture (PERVAP ™ 1210 type membrane) [74].

Pervaporation Units		Value	Unit
Permeate pressure		0.008	bar
Inlet pressure drop		0.1	bar
Permeability		10^8^	kmol/m^2^ hbar
Transport coefficient	Water	0.000202	kmol/m^2^ h
	Ethanol	0.0000193	
Activity energy	Water	77,877	kJ/kmol
	Ethanol	128,572	
Parameter “B”	Water	2.63	-
	Ethanol	−8.68	

**Table 3:** Organophilic pervaporation membrane parameters for ethanol-water mixture (PERVAP™ 4060 type membrane) [75].

Pervaporation Units		Value	Unit
Permeate pressure		0.008	bar
Inlet pressure drop		0.1	bar
Permeability		10^8^	kmol/m^2^ hbar
Transport coefficient	Water	0.026	kmol/m^2^ h
	Ethanol	0.077	
Activity energy	Water	31,363	kJ/kmol
	Ethanol	33,090	
Parameter “B”	Water	−0.73	-
	Ethanol	−0.04	

**Table 4:** Hydrophilic pervaporation membrane parameters for methanol-water mixture (PERVAP™ 1510 type membrane) [70].

Pervaporation Units		Value	Unit
Permeate pressure		0.008	bar
Inlet pressure drop		0.1	bar
Permeability		10^8^	kmol/m^2^ hbar
Transport coefficient	Water	0.167	kmol/m^2^ h
	Methanol	0.00018	
Activity energy	Water	23,498	kJ/kmol
	Methanol	30,795	
Parameter “B”	Water	−6.51	-
	Methanol	−2.4	

**Table 5:** Organophilic pervaporation membrane parameters of methanol-water mixture (PERVAP™ 4060 type membrane) [76].

Pervaporation Units		Value	Unit
Permeate pressure		0.008	bar
Inlet pressure drop		0.1	bar
Permeability		10^8^	kmol/m^2^ hbar
Transport coefficient	Water	0.00246	kmol/m^2^ h
	Methanol	0.0458	
Activity energy	Water	44,170	kJ/kmol
	Methanol	45,646	
Parameter “B“	Water	1.19	-
	Methanol	−5.64	

**Table 6:** Distillation column parameters.

Parameters	
Thermodynamic model	UNIQUAC
Column type	SCDS
Column material	Carbon steel
Plate type	Valve, SS304
Plate material	Carbon steel
Distillation product ethanol (or methanol)	target min. 0.9 m/m
Bottom product water	0.9999 m/m

**Table 7:** Dynamic tank parameters.

Parameters		Value	Unit
Dynamic tank	diameter	5	m
	cylinder height	10	m
	pressure	1	bar
	initial fluid level 1	2	m
	initial fluid level 2	10^−10^	m
**Time**		600	min

**Table 8:** Feed characteristics of hydrophilic and organophilic pervaporation membrane modelling.

Characteristics		Value	Unit
Feed pressure		1	bar
Feed temperature		20	°C
Feed flow		1000	kg/h
Feed composition	Water	0.98	m/m
	Ethanol (or methanol)	0.02	m/m

**Table 9:** Total heat consumptions of ethanol-water and methanol-water methods.

Methods	Total Heat Consumptions (MJ/h)					
	Ethanol-Water			Methanol-Water		
	1 × 40 m^2^	5 × 40 m^2^	10 × 40 m^2^	1 × 40 m^2^	5 × 40 m^2^	10 × 40 m^2^
OPV	−7.00	−177.73	−207.12	−7.47	−219.14	−248.12
HPV	−8.47	−37.54	−68.54	−0.34	−2.64	−3.29
Recirc HPV	−0.22	−0.28	−0.34	−0.21	−1.99	−2.00
Dyn OPV	−75763.30	−73057.06	−73389.00	−75904.13	−73030.71	−73247.39
Dyn HPV	−1.85 × 10^6^	−1.79 × 10^6^	−1.73 × 10^6^	−9.96 × 10^6^	−9.96 × 10^6^	−9.96 × 10^6^
D + OPV	−6.66	−173.07	−202.19	−7.25	−215.19	−243.90
D + HPV	325.86	325.65	325.55	325.83	324.36	324.24
Recirc D + HPV	326.30	326.71	326.63	326.32	325.41	325.32

## Supplementary Materials

The following are available online at https://www.mdpi.com/2077-0375/10/11/345/s1, Table S1: Results of OPV method for ethanol-water mixture, Table S2: Results of HPV method for ethanol-water mixture, Table S3: Results of Recirc HPV method for ethanol-water mixture., Table S4: Results of Dyn OPV method for ethanol-water mixture, Table S5: Results of Dyn HPV method for ethanol-water mixture, Table S6: Results of D + OPV method for ethanol-water mixture, Table S7: Results of D + HPV method for ethanol-water mixture, Table S8: Results of Recirc D + HPV method for ethanol-water mixture, Table S9: Results of OPV method for methanol-water mixture, Table S10: Results of HPV method for methanol-water mixture, Table S11: Results of Recirc HPV method for methanol-water mixture, Table S12: Results of Dyn OPV method for methanol-water mixture, Table S13: Results of Dyn HPV method for methanol-water mixture, Table S14: Results of D + OPV method for methanol-water mixture, Table S15: Results of D + HPV method for methanol-water mixture, Table S16: Results of Recirc D + HPV method for methanol-water mixture.

## Nomenclature

$J_{i}$	Partial flux $\left[kg/\left(m^{2}h\right)\right]$
${\bar{D}}_{i}$	Transport coefficient of component *i* $\left[kmol/\left(m^{2}h\right)\right]$
Q_0_	Permeability coefficient of the porous support layer of the membrane $\left[kmol/\left(m^{2}hbar\right)\right]$
$p_{i0}$	Pure i component vapour pressure [bar]
$p_{i1}$	Partial pressure of component i on the vapor phase membrane side [bar]
$p_{i3}$	Partial pressure of component i. on the vapour phase membrane side [bar]
${\bar{\gamma }}_{i}$	Average activity coefficient of component *i*
$x_{i1}$	Concentration of component i in the feed [m⁄(m%)]
$E_{i}$	Activation energy of component i. in Equation (1) for temperature dependence of the transport coefficient [kJ/mol]
B	Constant in pervaporation model [-]
