# Supplementary material for: Separation of Alcohol-Water Mixtures by a Combination of Distillation, Hydrophilic and Organophilic Pervaporation Processes
Source: Membranes (Basel). 2020 Nov 16;10(11):345. doi: 10.3390/membranes10110345 (PMC7697844; doi:10.3390/membranes10110345)
Supplement: Supplementary file 1 [file membranes-10-00345-s001.pdf]

Supplementary Materials

Separation of Alcohol-Water Mixtures by a Combination of Distillation, Hydrophilic and Organophilic Pervaporation Processes

Huyen Trang Do Thi [1], Peter Mizsey [1,2] and Andras Jozsef Toth [1,*]

[1] Environmental and Process Engineering Research Group, Department of Chemical and Environmental Process Engineering, Budapest University of Technology and Economics, Műegyetem rkp. 3, H-1111 Budapest, Hungary; dothihuyentrang@edu.bme.hu (H.T.D.T.); mizsey.miskolc@gmail.com (P.M.)

[2] Institute of Chemistry, University of Miskolc, Egyetemváros C/1 108, H-3515 Miskolc, Hungary

* Correspondence: andrasjozseftoth@edu.bme.hu; Tel.: +36-1-463-1490

Table S1. Results of OPV method for ethanol-water mixture

	Ethanol-water OPV	Unit	1. model	2. model	3. model	4. model	5. model	6. model	7. model	8. model	9. model	10. model
**Membrane surface**		m^2	40	40	40	40	40	40	40	40	40	40
**Section number**		-	400	400	400	400	400	400	400	400	400	400
**Pervaporation module**		piece	1	2	3	4	5	6	7	8	9	10
**Membrane area**		m^2	40	80	120	160	200	240	280	320	360	400
**Result**	Permeate water	m/m	0.885	0.898	0.914	0.922	0.929	0.935	0.94	0.945	0.949	0.952
	Permeate ethanol	m/m	0.115	0.102	0.086	0.078	0.071	0.065	0.06	0.055	0.051	0.048
	Permeate stream	kg/h	42.418	83.945	147.228	186.134	223.695	260.363	296.186	331.189	365.385	398.778
	Permeate temperature	°C	-20	-20	-20	-20	-20	-20	-20	-20	-20	-20
	Retentate water	m/m	0.984	0.987	0.991	0.993	0.995	0.996	0.997	0.997	0.998	0.998
	Retentate ethanol	m/m	0.016	0.013	0.009	0.007	0.005	0.004	0.003	0.003	0.002	0.002
	Retentate stream	kg/h	957.582	916.055	852.772	813.866	776.305	739.637	703.814	668.811	634.615	601.222
	Retentate temperature	°C	20	20	20	20	20	20	20	20	20	20
**Heat consumption**	Preheating	MJ/h	207.48	207.48	207.48	207.48	207.48	207.48	207.48	207.48	207.48	207.48
	Retentate heating	MJ/h	0.00	89.56	178.98	178.98	264.98	348.47	430.34	510.59	589.19	666.12
	Retentate cooling	MJ/h	-109.43	-101.16	-177.69	-83.70	-78.46	-72.50	-66.69	-61.06	-55.63	-50.39
	Permeate cooling	MJ/h	-105.05	-209.62	-373.80	-474.24	-571.73	-667.31	-760.99	-852.74	-942.54	-1030.33
	Total	MJ/h	-7.00	-13.75	-165.03	-171.48	-177.73	-183.86	-189.86	-195.74	-201.49	-207.12

Table S2. Results of HPV method for ethanol-water mixture

	Ethanol- water HPV	Unit	1. model	2. model	3. model	4. model	5. model	6. model	7. model	8. model	9. model	10. model
	**Membrane surface**	m²	40	40	40	40	40	40	40	40	40	40
	**Section number**	-	400	400	400	400	400	400	400	400	400	400
	**Pervaporation module**	piece	1	2	3	4	5	6	7	8	9	10
	**Membrane area**	m²	40	80	120	160	200	240	280	320	360	400
**Result**	Permeate water	m/m	0.998	0.998	0.998	0.998	0.998	0.998	0.998	0.998	0.998	0.998
	Permeate ethanol	m/m	0.002	0.002	0.002	0.002	0.002	0.002	0.002	0.002	0.002	0.002
	Permeate stream	kg/h	48.833	93.874	137.569	179.824	220.655	260.077	298.102	334.748	370.031	403.969
	Permeate temperature	°C	-20	-20	-20	-20	-20	-20	-20	-20	-20	-20
	Retentate water	m/m	0.979	0.978	0.977	0.976	0.975	0.974	0.972	0.971	0.969	0.968
	Retentate ethanol	m/m	0.021	0.022	0.023	0.024	0.025	0.026	0.028	0.029	0.031	0.032
	Retentate stream	kg/h	951.167	906.126	862.431	820.176	779.345	739.923	701.898	665.252	629.969	596.031
	Retentate temperature	°C	20	20	20	20	20	20	20	20	20	20
**Heat consumption**	Preheating	MJ/h	207.48	207.48	207.48	207.48	207.48	207.48	207.48	207.48	207.48	207.48
	Retentate heating	MJ/h	0.00	110.55	212.76	311.87	407.67	500.20	589.50	675.59	758.52	838.33
	Retentate cooling	MJ/h	-86.72	-85.66	-79.63	-74.12	-68.85	-63.86	-59.12	-54.63	-50.38	-46.37
	Permeate cooling	MJ/h	-129.22	-248.46	-364.09	-475.86	-583.83	-688.02	-788.49	-885.27	-978.42	-1067.97
	Total	MJ/h	-8.47	-16.09	-23.48	-30.63	-37.54	-44.20	-50.64	-56.83	-62.80	-68.54

Table S3. Results of Recirc HPV method for ethanol-water mixture

	Ethanol-water Recirc HPV	Unit	1. model	2. model	3. model	4. model	5. model	6. model	7. model	8. model	9. model	10. model
	**Membrane surface**	m²	40	40	40	40	40	40	40	40	40	40
	**Section number**	-	400	400	400	400	400	400	400	400	400	400
	**Pervaporation module**	piece	1	2	3	4	5	6	7	8	9	10
	**Membrane area**	m²	40	80	120	160	200	240	280	320	360	400
**Result**	Recycle Permeate water	m/m	0.998	0.998	0.998	0.998	0.998	0.998	0.999	0.999	0.999	0.999
	Recycle Permeate ethanol	m/m	0.002	0.002	0.002	0.002	0.002	0.002	0.001	0.001	0.001	0.001
	Recycle Permeate stream	kg/h	49.951	99.068	149.741	201.857	255.436	310.501	367.014	424.991	484.431	545.33
	Recycle Permeate temperature	°C	-20	-20	-20	-20	-20	-20	-20	-20	-20	-20
	Retentate water	m/m	0.980	0.98	0.980	0.98	0.98	0.98	0.98	0.98	0.98	0.98
	Retentate ethanol	m/m	0.020	0.02	0.020	0.02	0.02	0.02	0.02	0.02	0.02	0.02
	Retentate stream	kg/h	999.981	999.992	1000.01	999.935	999.882	999.971	999.951	999.926	999.89	999.85
	Retentate temperature	°C	20	20	20	20	20	20	20	20	20	20
**Heat consumption**	Preheating	MJ/h	226.36	244.93	264.10	283.77	304.01	324.87	346.23	368.14	390.60	413.62
	Retentate heating	MJ/h	0.00	116.62	231.58	350.11	471.98	597.29	725.93	857.96	993.36	1132.13
	Retentate cooling	MJ/h	-94.32	-99.34	-99.11	-99.11	-99.10	-99.12	-99.11	-99.11	-99.10	-99.10
	Permeate cooling	MJ/h	-132.26	-262.45	-396.80	-535.03	-677.17	-823.31	-973.34	-1127.29	-1285.18	-1446.99
	Total	MJ/h	-0.22	-0.23	-0.24	-0.26	-0.28	-0.27	-0.29	-0.30	-0.32	-0.34

Table S4. Results of Dyn OPV method for ethanol-water mixture

Ethanol-water Dyn OPV		Unit	1. model	2. model	3. model	4. model	5. model	6. model	7. model	8. model	9. model	10. model
**Membrane surface**		m²	40	40	40	40	40	40	40	40	40	40
**Section number**		-	400	400	400	400	400	400	400	400	400	400
**Pervaporation module**		piece	1	2	3	4	5	6	7	8	9	10
**Membrane area**		m²	40	80	120	160	200	240	280	320	360	400
**Permeate Recycle**	Water	m/m	0.852	0.834	0.808	0.852	0.909	0.939	0.956	0.966	0.975	0.979
	Ethanol	m/m	0.148	0.166	0.192	0.148	0.091	0.061	0.044	0.034	0.025	0.021
	Stream	kg/h	44.329	91.681	142.286	182.245	214.838	249.721	285.341	321.153	356.524	392.366
**Retentate**	Water	m/m	0.98	0.98	0.98	0.986	0.9921	0.9952	0.997	0.998	0.99868	0.99905
	Ethanol	m/m	0.02	0.02	0.02	0.014	0.0079	0.0048	0.003	0.002	0.00132	0.00095
	Stream	kg/h	999.842	999.981	999.964	977.481	940.144	903.688	867.870	832.574	797.927	763.588
**Heat consumption**	1. dynamic tank	MJ/h	-7.57E+04	-7.43E+04	-7.26E+04	-7.25E+04	-7.31E+04	-7.34E+04	-7.37E+04	-7.37E+04	-7.39E+04	-7.39E+04
	Retentate heating	MJ/h	155.70	113.97	113.94	159.41	241.87	325.88	410.31	494.51	577.86	660.88
	Retentate cooling	MJ/h	-192.73	-191.04	-191.01	-184.05	-174.06	-164.41	-155.24	-146.41	-137.89	-129.59
	2. dynamic tank	MJ/h	-26.27	-26.27	-26.27	-25.77	-24.87	-23.95	-23.03	-22.10	-21.19	-20.29
	Total	MJ/h	-75763.30	-74403.34	-72703.34	-72550.40	-73057.06	-73262.48	-73467.95	-73374.00	-73481.22	-73389.00

Table S5. Results of Dyn HPV method for ethanol-water mixture

Ethanol-water Dyn HPV		Unit	1. model	2. model	3. model	4. model	5. model	6. model	7. model	8. model	9. model	10. model
**Membrane surface**		m²	40	40	40	40	40	40	40	40	40	40
**Section number**		-	400	400	400	400	400	400	400	400	400	400
**Pervaporation module**		piece	1	2	3	4	5	6	7	8	9	10
**Membrane area**		m²	40	80	120	160	200	240	280	320	360	400
**Permeate**	Water	m/m	0.995	0.995	0.995	0.995	0.995	0.995	0.995	0.995	0.995	0.995
	Ethanol	m/m	0.005	0.005	0.005	0.005	0.005	0.005	0.005	0.005	0.005	0.005
	Stream	kg/h	24.63	47.958	70.207	91.489	110.817	131.109	148.372	166.051	178.998	198.801
**Recycle**	Water	m/m	0.223	0.22	0.216	0.213	0.208	0.206	0.202	0.199	0.193	0.193
	Ethanol	m/m	0.777	0.78	0.784	0.787	0.792	0.794	0.798	0.801	0.807	0.807
	Stream	kg/h	1.07E+07	1.06E+06	1.06E+07	1.05E+07	1.05E+07	1.04E+07	1.04E+07	1.03E+07	1.03E+07	1.03E+07
**Heat consumption**	1. dynamic vessel	MJ/h	-1.85E+06	-1.83E+06	-1.82E+06	-1.80E+06	-1.79E+06	-1.78E+06	-1.76E+06	-1.75E+06	-1.73E+06	-1.73E+06
	Precooling	MJ/h	-2467.23	251.143	254.998	247.984	250.946	247.377	247.201	251.614	249.063	248.856
	Retentate heating	MJ/h	0	-3067.7	-3001.136	-2918.773	-2854.918	-2815.042	-2754.703	-2671.079	-2298.583	-2604.493
	Retentate cooling	MJ/h	-63.90	-124.43	-182.17	-237.39	-287.55	-340.21	-385.02	-430.90	-464.52	-515.91
	2. dynamic tank	MJ/h	1.00E-10	1.00E-10	1.00E-10	1.00E-10	1.00E-10	1.00E-10	1.00E-10	1.00E-10	1.00E-10	1.00E-10
	Total	MJ/h	-1847531.1	-1832941	-1818928.3	-1805908	-1787891.5	-1779907.9	-1762892.5	-1751850.4	-1732514	-1728871.6

Table S6. Results of D+OPV method for ethanol-water mixture

**Ethanol-water D+OPV**			**Unit**	**1. model**	**2. model**	**3. model**	**4. model**	**5. model**	**6. model**	**7. model**	**8. model**	**9. model**	**10. model**
**Membrane surface**			m^2	40	40	40	40	40	40	40	40	40	40
**Section number**			-	400	400	400	400	400	400	400	400	400	400
**Pervaporation module**			piece	1	2	3	4	5	6	7	8	9	10
**Membrane area**			m^2	40	80	120	160	200	240	280	320	360	400
**Result**	Bottom product	Permeate water	m/m	0.9944	0.995	0.9956	0.9961	0.9964	0.9967	0.997	0.9972	0.9974	0.9976
		Permeate ethanol	m/m	0.0056	0.005	0.0044	0.0039	0.0036	0.0033	0.003	0.0028	0.0026	0.0024
		Permeate stream	kg/h	38.202	76.587	135.885	173.229	209.583	245.315	280.41	314.845	348.596	381.637
		Permeate temperature	°C	-20	-20	-20	-20	-20	-20	-20	-20	-20	-20
		Retentate water	m/m	0.99919	0.99934	0.99954	0.99963	0.99971	0.99977	0.99982	0.99986	0.99989	0.999915
		Retentate ethanol	m/m	0.00081	0.00066	0.00046	0.00037	0.00029	0.00023	0.00018	0.00014	0.00011	8.5E-05
		Retentate stream	kg/h	940.663	902.279	842.98	805.637	769.282	733.55	698.455	664.02	630.269	597.229
		Retentate temperature	°C	20	20	20	20	20	20	20	20	20	20
	Distillate	Water	m/m	0.1	0.1	0.1	0.1	0.1	0.1	0.1	0.1	0.1	0.1
		Ethanol	m/m	0.9	0.9	0.9	0.9	0.9	0.9	0.9	0.9	0.9	0.9
		Current	kg/h	21.135	21.135	21.135	21.135	21.135	21.135	21.135	21.135	21.135	21.135
		Temperature	°C	20	20	20	20	20	20	20	20	20	20
**Heat consumption**		Preheating	MJ/h	-121.47	-121.47	-121.47	-121.47	-121.47	-121.47	-121.47	-121.47	-121.47	-121.47
		Bottom product cooling	MJ/h	-24.33	-24.33	-24.33	-24.33	-24.33	-24.33	-24.33	-24.33	-24.33	-24.33
		Retentate heating	MJ/h	0.00	86.85	174.10	174.10	258.95	341.55	422.69	502.36	580.48	657.01
		Retentate cooling	MJ/h	-109.72	-101.30	-176.18	-83.53	-78.19	-72.17	81.15	-60.67	-55.21	-49.96
		Permeate cooling	MJ/h	-101.27	-203.02	-362.86	-461.82	-558.16	-652.82	-745.75	-836.90	-926.19	-1013.56
		Distillation	MJ/h	-385.23	-385.23	-385.23	-385.23	-385.23	-385.23	-385.23	-385.23	-385.23	-385.23
			MJ/h	735.36	735.36	735.36	735.36	735.36	735.36	735.36	735.36	735.36	735.36
		Heating sum	MJ/h	613.89	700.74	787.99	787.99	872.84	955.43	1036.58	1116.25	1194.37	1270.90
		Cooling sum	MJ/h	-620.55	-713.88	-948.60	-954.91	-1045.91	-1134.55	-1074.17	-1307.13	-1390.97	-1473.08
		Total	MJ/h	-6.66	-13.14	-160.61	-166.92	-173.07	-179.12	-37.59	-190.88	-196.60	-202.19

Table S7. Results of D+HPV method for ethanol-water mixture

Ethanol- water D+HPV			Unit	1. model	2. model	3. model	4. model	5. model	6. model	7. model	8. model	9. model	10. model
**Membrane surface**			m²	40	40	40	40	40	40	40	40	40	40
**Section number**			-	400	400	400	400	400	400	400	400	400	400
**Pervaporation module**			piece	1	2	3	4	5	6	7	8	9	10
**Membrane area**			m²	40	80	120	160	200	240	280	320	360	400
**Result**	Distillate	Permeate water	m/m	0.998	0.996	0.995	0.992	0.989	0.985	0.981	0.977	0.974	0.97
		Permeate ethanol	m/m	0.002	0.004	0.005	0.008	0.011	0.015	0.019	0.023	0.026	0.03
		Permeate stream	kg/h	0.888	1.548	1.955	2.118	2.163	2.179	2.187	2.197	2.205	2.213
		Permeate temperature	°C	-20	-20	-20	-20	-20	-20	-20	-20	-20	-20
		Retentate water	m/m	0.063	0.033	0.014	0.006	0.004	0.004	0.004	0.004	0.004	0.004
		Retentate ethanol	m/m	0.937	0.967	0.986	0.994	0.996	0.996	0.996	0.996	0.996	0.996
		Retentate stream	kg/h	21.324	20.663	20.257	20.093	20.048	20.032	20.028	20.018	20.012	20.004
		Retentate temperature	°C	20	20	20	20	20	20	20	20	20	20
	Bottom product	Water	m/m	0.99999	0.99999	0.99999	0.99999	0.99999	0.99999	0.99999	0.99999	0.99999	0.99999
		Ethanol	m/m	1E-05	1E-05	1E-05	1E-05	1E-05	1E-05	1E-05	1E-05	1E-05	1E-05
		Current	kg/h	977.788	977.789	977.788	977.789	977.789	977.789	977.785	977.785	977.783	977.783
		Temperature	°C	99.6	99.6	99.6	99.6	99.6	99.6	99.6	99.6	99.6	99.6
**Heat consumption**		Preheating	MJ/h	3.09	3.09	3.09	3.09	3.09	3.09	3.09	3.09	3.09	3.09
		Precooling	MJ/h	-25.57	-25.57	-25.57	-25.57	-25.57	-25.57	-25.57	-25.57	-25.57	-25.57
		Retentate heating	MJ/h	0.00	2.00	3.50	4.43	4.80	4.89	4.91	4.90	4.91	4.89
		Retentate cooling	MJ/h	-0.91	-1.27	-1.76	-2.28	-2.55	-2.62	-2.65	-2.63	-2.66	-2.64
		Permeate cooling	MJ/h	-2.34	-4.09	-5.16	-5.59	-5.70	-5.73	-5.78	-5.79	-5.80	-5.81
		Distillation	MJ/h	-2222.91	-2222.91	-2222.91	-2222.91	-2222.91	-2222.91	-2222.91	-2222.91	-2222.91	-2222.91
			MJ/h	2574.50	2574.50	2574.50	2574.50	2574.50	2574.50	2574.50	2574.50	2574.50	2574.50
		Heating sum	MJ/h	2577.59	2579.59	2581.09	2582.01	2582.38	2582.48	2582.50	2582.49	2582.50	2582.48
		Cooling sum	MJ/h	-2251.73	-2253.84	-2255.41	-2256.36	-2256.74	-2256.83	-2256.91	-2256.90	-2256.94	-2256.93
		Total	MJ/h	325.86	325.75	325.68	325.65	325.65	325.64	325.59	325.58	325.56	325.55

Table S8. Results of Recirc D+HPV method for ethanol-water mixture

Ethanol-water Recirc D+HPV			Unit	1. model	2. model	3. model	4. model	5. model	6. model	7. model	8. model	9. model	10. model
**Membrane surface**			m²	40	40	40	40	40	40	40	40	40	40
**Section number**			-	400	400	400	400	400	400	400	400	400	400
**Pervaporation module**			piece	1	2	3	4	5	6	7	8	9	10
**Membrane area**			m²	40	80	120	160	200	240	280	320	360	400
**Result**	Bottom product	recir. Permeate water	m/m	0.998	0.996	0.995	0.992	0.989	0.985	0.981	0.977	0.974	0.970
		recir. Permeate ethanol	m/m	0.002	0.004	0.005	0.008	0.011	0.015	0.019	0.023	0.026	0.03
		recir. Permeate stream	kg/h	0.888	1.548	1.955	2.12	2.166	2.183	2.191	2.202	2.211	2.221
		recir. Permeate temperature	°C	-20	-20	-20	-20	-20	-20	-20	-20	-20	-20
		Retentate water	m/m	0.063	0.033	0.014	0.006	0.004	0.004	0.004	0.004	0.004	0.004
		Retentate ethanol	m/m	0.937	0.967	0.986	0.994	0.996	0.996	0.996	0.996	0.996	0.996
		Retentate stream	kg/h	21.326	20.669	20.267	20.109	20.073	20.066	20.069	20.068	20.069	20.07
		Retentate temperature	°C	20	20	20	20	20	20	20	20	20	20
	Distillate	Water	m/m	0.99999	0.99999	0.99999	0.99999	0.99999	0.99999	0.99999	0.99999	0.99999	0.99999
		Ethanol	m/m	1E-05	1E-05	1E-05	1E-05	1E-05	1E-05	1E-05	1E-05	1E-05	1E-05
		Current	kg/h	978.674	979.331	979.733	979.891	979.927	979.934	979.934	979.936	979.936	979.936
		Temperature	°C	99.6	99.6	99.6	99.6	99.6	99.6	99.6	99.6	99.6	99.6
**Heat consumption**		Preheating	MJ/h	3.09	3.09	3.09	3.09	3.09	3.09	3.10	3.10	3.10	3.10
		Precooling	MJ/h	-25.58	-25.58	-25.59	-25.60	-25.61	-25.62	-25.63	-25.64	-25.65	-25.66
		Retentate heating	MJ/h	0.00	2.00	3.50	4.43	4.80	4.90	4.92	4.91	4.93	4.90
		Retentate cooling	MJ/h	-0.91	-1.27	-1.76	-2.28	-2.55	-2.62	-2.66	-2.64	-2.67	-2.64
		Permeate cooling	MJ/h	-2.34	-4.09	-5.17	-5.60	-5.71	-5.74	-5.79	-5.81	-5.82	-5.83
		Distillation	MJ/h	-2225.90	-2237.07	-2230.50	-2240.95	-2239.57	-2230.26	-2231.43	-2226.96	-2229.95	-2240.11
			MJ/h	2577.93	2589.45	2583.07	2593.61	2592.26	2582.98	2584.15	2579.70	2582.67	2592.87
		Heating sum	MJ/h	2581.02	2594.54	2589.66	2601.13	2600.15	2590.97	2592.17	2587.71	2590.69	2600.87
		Cooling sum	MJ/h	-2254.72	-2268.01	-2263.01	-2274.43	-2273.44	-2264.24	-2265.50	-2261.04	-2264.08	-2274.24
		Total	MJ/h	326.30	326.53	326.65	326.70	326.71	326.73	326.66	326.67	326.61	326.63

Table S9. Results of OPV method for methanol-water mixture

	Methanol-water OPV	Unit	1. model	2. model	3. model	4. model	5. model	6. model	7. model	8. model	9. model	10. model
	**Membrane surface**	m²	40	40	40	40	40	40	40	40	40	40
	**Section number**	-	400	400	400	400	400	400	400	400	400	400
	**Pervaporation module**	piece	1	2	3	4	5	6	7	8	9	10
	**Membrane area**	m²	40	80	120	160	200	240	280	320	360	400
**Result**	Permeate water	m/m	0.9	0.907	0.921	0.927	0.933	0.938	0.943	0.947	0.95	0.954
	Permeate methanol	m/m	0.1	0.093	0.079	0.073	0.067	0.062	0.057	0.053	0.05	0.046
	Permeate stream	kg/h	44.812	87.066	166.719	204.397	241.96	278.446	313.912	348.38	381.865	414.375
	Permeate temperature	°C	-20	-20	-20	-20	-20	-20	-20	-20	-20	-20
	Retentate water	m/m	0.984	0.987	0.992	0.994	0.995	0.996	0.997	0.998	0.998	0.999
	Retentate methanol	m/m	0.016	0.013	0.008	0.006	0.005	0.004	0.003	0.002	0.002	0.001
	Retentate stream	kg/h	955.188	912.934	833.281	795.603	758.04	721.554	686.088	651.62	618.135	585.625
	Retentate temperature	°C	20	20	20	20	20	20	20	20	20	20
**Heat consumption**	Preheating	MJ/h	207.52	207.52	207.52	207.52	207.52	207.52	207.52	207.52	207.52	207.52
	Retentate heating	MJ/h	0.00	96.72	188.80	188.80	272.55	356.45	438.22	517.90	595.48	670.95
	Retentate cooling	MJ/h	-101.77	-97.85	-173.66	-82.17	-74.26	-68.85	-63.58	-58.51	-53.66	-49.01
	Permeate cooling	MJ/h	-113.22	-220.75	-429.28	-527.03	-624.95	-720.37	-813.36	-903.90	-991.98	-1077.58
	Total	MJ/h	-7.47	-14.36	-206.63	-212.88	-219.14	-225.25	-231.20	-236.99	-242.64	-248.12

Table S10. Results of HPV method for methanol-water mixture

	Methanol- water HPV	Unit	1. model	2. model	3. model	4. model	5. model	6. model	7. model	8. model	9. model	10. model
	**Membrane surface**	m²	40	40	40	40	40	40	40	40	40	40
	**Section number**	-	400	400	400	400	400	400	400	400	400	400
	**Pervaporation module**	piece	1	2	3	4	5	6	7	8	9	10
	**Membrane area**	m²	40	80	120	160	200	240	280	320	360	400
**Result**	Permeate water	m/m	0.979	0.979	0.979	0.979	0.979	0.979	0.979	0.979	0.979	0.98
	Permeate methanol	m/m	0.021	0.021	0.021	0.021	0.021	0.021	0.021	0.021	0.021	0.02
	Permeate stream	kg/h	0.77	1.542	2.319	3.092	3.864	4.637	5.409	6.182	6.954	7.727
	Permeate temperature	°C	-20	-20	-20	-20	-20	-20	-20	-20	-20	-20
	Retentate water	m/m	0.980	0.98	0.980	0.98	0.98	0.98	0.98	0.98	0.98	0.98
	Retentate methanol	m/m	0.020	0.02	0.020	0.02	0.020	0.02	0.020	0.02	0.020	0.02
	Retentate stream	kg/h	999.23	998.458	997.681	996.908	996.136	995.363	994.591	993.818	993.046	992.273
	Retentate temperature	°C	20	20	20	20	20	20	20	20	20	20
**Heat consumption**	Preheating	MJ/h	207.52	207.52	207.52	207.52	207.52	207.52	207.52	207.52	207.52	207.52
	Retentate heating	MJ/h	0.00	1.56	3.34	3.34	5.12	6.89	8.67	10.44	12.22	14.00
	Retentate cooling	MJ/h	-205.80	-205.42	-207.04	-205.10	-204.94	-204.78	-204.62	-204.46	-204.30	-204.14
	Permeate cooling	MJ/h	-2.06	-4.12	-6.20	-8.27	-10.33	-12.40	-14.46	-16.53	-18.59	-20.66
	Total	MJ/h	-0.34	-0.47	-2.38	-2.51	-2.64	-2.77	-2.90	-3.03	-3.16	-3.29

Table S11. Results of Recirc HPV method for methanol-water mixture

Methanol- water Recirc HPV		Unit	1. model	2. model	3. model	4. model	5. model	6. model	7. model	8. model	9. model	10. model
**Membrane surface**		m²	40	40	40	40	40	40	40	40	40	40
**Section number**		-	400	400	400	400	400	400	400	400	400	400
**Pervaporation module**		piece	1	2	3	4	5	6	7	8	9	10
**Membrane area**		m²	40	80	120	160	200	240	280	320	360	400
**Result**	Recycle Permeate water	m/m	0.979	0.979	0.979	0.979	0.979	0.979	0.979	0.979	0.979	0.979
	Recycle Permeate methanol	m/m	0.021	0.021	0.021	0.021	0.021	0.021	0.021	0.021	0.021	0.021
	Recycle Permeate stream	kg/h	0.77	1.542	2.319	3.092	3.864	4.637	5.409	6.182	6.954	7.727
	Recycle Permeate temperature	°C	-20	-20	-20	-20	-20	-20	-20	-20	-20	-20
	Retentate water	m/m	0.980	0.98	0.980	0.98	0.98	0.98	0.98	0.98	0.98	0.98
	Retentate methanol	m/m	0.020	0.02	0.020	0.02	0.020	0.02	0.020	0.02	0.020	0.02
	Retentate stream	kg/h	1000	1000	1000	1000	1000	1000	1000	1000	1000	1000
	Retentate temperature	°C	20	20	20	20	20	20	20	20	20	20
**Heat consumption**	Preheating	MJ/h	207.81	208.10	208.39	208.68	208.97	209.26	209.55	209.84	210.13	210.42
	Retentate heating	MJ/h	0.00	1.56	3.34	3.34	5.12	6.89	8.66	10.44	12.21	13.99
	Retentate cooling	MJ/h	-205.96	-205.74	-207.52	-205.74	-205.74	-205.74	-205.74	-205.74	-205.74	-205.74
	Permeate cooling	MJ/h	-2.06	-4.12	-6.20	-8.27	-10.33	-12.40	-14.46	-16.53	-18.59	-20.66
	Total	MJ/h	-0.21	-0.21	-1.99	-1.99	-1.99	-1.99	-1.99	-1.99	-2.00	-2.00

Table S12. Results of Dyn OPV method for methanol-water mixture

Methanol-water Dyn OPV		Unit	1. model	2. model	3. model	4. model	5. model	6. model	7. model	8. model	9. model	10. model
**Membrane surface**		m²	40	40	40	40	40	40	40	40	40	40
**Section number**		-	400	400	400	400	400	400	400	400	400	400
**Pervaporation module**		piece	1	2	3	4	5	6	7	8	9	10
**Membrane area**		m²	40	80	120	160	200	240	280	320	360	400
**Permeate Recycle**	Water	m/m	0.88	0.864	0.85	0.872	0.904	0.927	0.944	0.956	0.965	0.971
	Methanol	m/m	0.12	0.136	0.15	0.128	0.096	0.073	0.056	0.044	0.035	0.029
	Stream	kg/h	46.336	93.193	141.887	185.314	224.331	262.47	300.035	337.104	373.645	409.591
**Retentate**	Water	m/m	0.98	0.98	0.98	0.985	0.991	0.994	0.9961	0.9974	0.9983	0.9988
	Methanol	m/m	0.02	0.02	0.02	0.015	0.009	0.006	0.0039	0.0026	0.0017	0.0012
	Stream	kg/h	999.843	999.977	999.967	971.793	931.791	893.44	856.26	819.99	784.504	749.92
**Heat consumption**	1. dynamic tank	MJ/h	- 7.58E+04	- 7.45E+04	- 7.30E+04	- 7.28E+04	- 7.31E+04	- 7.34E+04	- 7.35E+04	- 7.37E+04	- 7.37E+04	- 7.38E+04
	Retentate heating	MJ/h	108.34	110.80	110.73	172.66	263.26	352.60	440.79	527.79	613.46	697.73
	Retentate cooling	MJ/h	-186.15	-188.65	-188.59	-180.36	-169.32	-159.36	-150.14	-141.43	-133.13	-125.20
	2. dynamic tank	MJ/h	-26.32	-26.32	26.32	-25.64	-24.65	-23.68	-22.72	-21.77	-20.84	-19.92
	Total	MJ/h	-75904.13	-74604.17	-73051.55	-72833.34	-73030.71	-73230.44	-73232.06	-73335.41	-73240.50	-73247.39

Table S13. Results of Dyn HPV method for methanol-water mixture

Methanol-water Dyn HPV		Unit	1. model	2. model	3. model	4. model	5. model	6. model	7. model	8. model	9. model	10. model
**Membrane surface**		m^2	40	40	40	40	40	40	40	40	40	40
**Section number**		-	400	400	400	400	400	400	400	400	400	400
**Pervaporation module**		piece	1	2	3	4	5	6	7	8	9	10
**Membrane area**		m^2	40	80	120	160	200	240	280	320	360	400
**Permeate**	Water	m/m	0.9995	0.9995	0.9995	0.9995	0.9995	0.9995	0.9996	0.9996	0.9996	0.9996
	Methanol	m/m	0.0005	0.0005	0.0005	0.0005	0.0005	0.0005	0.0004	0.0004	0.0004	0.0004
	Stream	kg/h	0.713	1.426	2.138	2.851	3.563	4.275	4.987	5.699	6.411	7.123
**Recycle**	Water	m/m	0.9996	0.9996	0.9996	0.9996	0.9996	0.9996	0.9996	0.9996	0.9996	0.9996
	Methanol	m/m	0.0004	0.0004	0.0004	0.0004	0.0004	0.0004	0.0004	0.0004	0.0004	0.0004
	Stream	kg/h	3.52E+07	3.52E+07	3.52E+07	3.52E+07	3.52E+07	3.52E+07	3.52E+07	3.52E+07	3.52E+07	3.52E+07
**Heat consumption**	1. dynamic tank	MJ/h	-9.96E+06	-9.96E+06	-9.96E+06	-9.96E+06	-9.96E+06	-9.96E+06	-9.96E+06	-9.96E+06	-9.95E+06	-9.95E+06
	Precooling	MJ/h	-7084.15	189.932	177.759	160.461	189.932	188.46	184.275	179.874	179.106	176.743
	Retentate heating	MJ/h	0	-7262.06	-7250.39	-7250.91	-7267.75	-7276.46	-7287.91	-7306.15	-7322.898	-7355.37
	Retentate cooling	MJ/h	-1.86	-3.71	-5.56	-7.41	-9.26	-11.12	-12.97	-14.82	-16.67	-18.52
	2. dynamic tank	MJ/h	1.00E-10	1.00E-10	1.00E-10	1.00E-10	1.00E-10	1.00E-10	1.00E-10	1.00E-10	1.00E-10	1.00E-10
	Total	MJ/h	-9.96E+06	-9.96E+06	-9.96E+06	-9.96E+06	-9.96E+06	-9.96E+06	-9.96E+06	-9.97E+06	-9.96E+06	-9.96E+06

Table S14. Results of D+OPV method for methanol-water mixture

Methanol-water D+OPV			Unit	1. model	2. model	3. model	4. model	5. model	6. model	7. model	8. model	9. model	10. model
**Membrane surface**			m²	40	40	40	40	40	40	40	40	40	40
**Section number**			-	400	400	400	400	400	400	400	400	400	400
**Pervaporation module**			piece	1	2	3	4	5	6	7	8	9	10
**Membrane area**			m²	40	80	120	160	200	240	280	320	360	400
**Result**	Bottom product	Permeate water	m/m	0.995	0.997	0.996	0.997	0.997	0.997	0.997	0.997	0.997	0.998
		Permeate methanol	m/m	0.005	0.003	0.004	0.003	0.003	0.003	0.003	0.003	0.003	0.002
		Permeate stream	kg/h	41.695	81.308	157.26	193.674	230.247	265.938	300.762	334.705	367.754	399.895
		Permeate temperature	°C	-20	-20	-20	-20	-20	-20	-20	-20	-20	-20
		Retentate water	m/m	0.9992	0.99918	0.99949	0.9996	0.999692	0.99976	0.999817	0.99986	0.99989	0.99992
		Retentate methanol	m/m	0.0008	0.00082	0.00051	0.0004	0.000308	0.00024	0.000183	0.00014	0.00011	8E-05
		Retentate stream	kg/h	937.171	897.558	821.605	785.191	748.618	712.927	678.103	644.16	611.112	578.97
		Retentate temperature	°C	20	20	20	20	20	20	20	20	20	20
	Distillate	Water	m/m	0.1	0.1	0.1	0.1	0.1	0.1	0.1	0.1	0.1	0.1
		Methanol	m/m	0.9	0.9	0.9	0.9	0.9	0.9	0.9	0.9	0.9	0.9
		Stream	kg/h	21.134	21.134	21.135	21.135	21.135	21.135	21.135	21.135	21.134	21.135
		Temperature	°C	20	20	20	20	20	20	20	20	20	20
**Heat consumption**		Preheating	MJ/h	-121.52	-121.52	-121.52	-121.52	-121.52	-121.52	-121.52	-121.52	-121.52	-121.52
		Bottom product cooling	MJ/h	-28.73	-28.73	-28.73	-28.73	-28.73	-28.73	-28.73	-28.73	-28.73	-28.73
		Retentate heating	MJ/h	0.00	94.71	184.91	184.91	267.65	350.70	431.71	510.73	587.72	662.64
		Retentate cooling	MJ/h	-101.13	-97.35	-171.71	-81.36	-73.42	-67.99	-62.71	-57.65	-52.81	-48.19
		Permeate cooling	MJ/h	-110.45	-215.64	-420.38	-516.88	-613.75	-708.26	-800.45	-890.27	-977.70	-1062.68
		Distillation	MJ/h	-293.70	-293.70	-293.70	-293.70	-293.70	-293.70	-293.70	-293.70	-293.70	-293.70
			MJ/h	648.28	648.28	648.28	648.28	648.28	648.28	648.28	648.28	648.28	648.28
		Heating sum	MJ/h	526.76	621.46	711.67	711.67	794.41	877.45	958.47	1037.49	1114.48	1189.39
		Cooling sum	MJ/h	-534.00	-635.42	-914.51	-920.67	-1009.60	-1098.68	-1185.59	-1270.35	-1352.93	-1433.29
		Total	MJ/h	-7.25	-13.95	-202.84	-209.00	-215.19	-221.23	-227.12	-232.86	-238.46	-243.90

Table S15. Results of D+HPV method for methanol-water mixture

**Methanol- water D+HPV**			**Unit**	**1. model**	**2. model**	**3. model**	**4. model**	**5. model**	**6. model**	**7. model**	**8. model**	**9. model**	**10. model**
**Membrane surface**			m^2	40	40	40	40	40	40	40	40	40	40
**Section number**			-	400	400	400	400	400	400	400	400	400	400
**Pervaporation module**			piece	1	2	3	4	5	6	7	8	9	10
**Membrane area**			m^2	40	80	120	160	200	240	280	320	360	400
**Result**	Distillate	Permeate water	m/m	0.969	0.951	0.911	0.871	0.839	0.809	0.781	0.755	0.731	0.708
		Permeate methanol	m/m	0.031	0.049	0.089	0.129	0.161	0.191	0.219	0.245	0.269	0.292
		Permeate stream	kg/h	1.035	1.731	2.269	2.373	2.464	2.556	2.647	2.738	2.829	2.921
		Permeate temperature	°C	-20	-20	-20	-20	-20	-20	-20	-20	-20	-20
		Retentate water	m/m	0.058	0.028	0.008	0.008	0.008	0.008	0.009	0.009	0.009	0.009
		Retentate methanol	m/m	0.942	0.972	0.992	0.992	0.992	0.992	0.991	0.991	0.991	0.991
		Retentate stream	kg/h	21.176	20.481	19.942	19.845	19.756	19.667	19.578	19.487	19.396	19.305
		Retentate temperature	°C	20	20	20	20	20	20	20	20	20	20
	Bottom product	Water	m/m	0.99999	0.99999	0.99999	0.99999	0.99999	0.99999	0.99999	0.99999	0.99999	0.99999
		Methanol	m/m	1E-05	1E-05	1E-05	1E-05	1E-05	1E-05	1E-05	1E-05	1E-05	1E-05
		Stream	kg/h	977.789	977.789	977.789	977.789	977.789	977.789	977.789	977.789	977.789	977.789
		Temperature	°C	99.6	99.6	99.6	99.6	99.6	99.6	99.6	99.6	99.6	99.6
**Heat consumption**		Preheating	MJ/h	3.13	3.13	3.13	3.13	3.13	3.13	3.13	3.13	3.13	3.13
		Precooling	MJ/h	-30.19	-30.19	-30.19	-30.19	-30.19	-30.19	-30.19	-30.19	-30.19	-30.19
		Retentate heating	MJ/h	0.00	2.29	3.82	3.82	3.82	3.90	3.97	4.04	4.14	4.25
		Retentate cooling	MJ/h	-0.63	-1.26	-2.68	-2.64	-2.57	-2.57	-2.56	-2.51	-2.50	-2.49
		Permeate cooling	MJ/h	-2.68	-4.46	-5.76	-5.90	-6.03	-6.16	-6.29	-6.41	-6.54	-6.67
		Distillation	MJ/h	-1549.18	-1549.18	-1549.18	-1549.18	-1549.18	-1549.18	-1549.18	-1549.18	-1549.18	-1549.18
			MJ/h	1905.38	1905.38	1905.38	1905.38	1905.38	1905.38	1905.38	1905.38	1905.38	1905.38
		Heating sum	MJ/h	1908.51	1910.81	1912.33	1912.33	1912.33	1912.41	1912.48	1912.55	1912.66	1912.76
		Cooling sum	MJ/h	-1582.68	-1585.09	-1587.81	-1587.92	-1587.97	-1588.10	-1588.22	-1588.30	-1588.41	-1588.53
		Total	MJ/h	325.83	325.72	324.52	324.41	324.36	324.31	324.26	324.25	324.24	324.24

Table S16. Results of Recirc D+HPV method for methanol-water mixture

		Methanol-water Recirc D+HPV	Unit	1. model	2. model	3. model	4. model	5. model	6. model	7. model	8. model	9. model	10. model
		**Membrane surface**	m²	40	40	40	40	40	40	40	40	40	40
		**Section number**	-	400	400	400	400	400	400	400	400	400	400
		**Pervaporation module**	piece	1	2	3	4	5	6	7	8	9	10
		**Membrane area**	m²	40	80	120	160	200	240	280	320	360	400
**Result**	Bottom product	recir. Permeate water	m/m	0.969	0.952	0.912	0.874	0.842	0.813	0.785	0.761	0.737	0.716
		recir. Permeate methanol	m/m	0.031	0.048	0.088	0.126	0.158	0.187	0.215	0.239	0.263	0.284
		recir. Permeate stream	kg/h	1.036	1.737	2.288	2.401	2.503	2.607	2.699	2.807	2.898	3.003
		recir. Permeate temperature	°C	-20	-20	-20	-20	-20	-20	-20	-20	-20	-20
		Retentate water	m/m	0.058	0.028	0.008	0.008	0.008	0.008	0.008	0.008	0.009	0.008
		Retentate methanol	m/m	0.942	0.972	0.992	0.992	0.992	0.992	0.992	0.992	0.991	0.992
		Retentate stream	kg/h	21.21	20.568	20.146	20.154	20.157	20.16	20.07	20.16	20.074	20.073
		Retentate temperature	°C	20	20	20	20	20	20	20	20	20	20
	Distillate	Water	m/m	0.99999	0.99999	0.99999	0.99999	0.99999	0.99999	0.99999	0.99999	0.99999	0.99999
		Methanol	m/m	1E-05	1E-05	1E-05	1E-05	1E-05	1E-05	1E-05	1E-05	1E-05	1E-05
		Current	kg/h	978.79	979.432	979.854	979.852	979.852	979.853	979.94	979.85	979.938	979.942
		Temperature	°C	99.6	99.6	99.6	99.6	99.6	99.6	99.6	99.6	99.6	99.6
**Heat consumption**		Preheating	MJ/h	3.14	3.15	3.16	3.18	3.19	3.21	3.21	3.24	3.24	3.25
		Precooling	MJ/h	-30.24	-30.32	-30.50	-30.65	-30.79	-30.92	-30.94	-31.20	-31.21	-31.35
		Retentate heating	MJ/h	0.00	2.30	3.85	3.87	3.91	3.96	4.11	4.21	4.33	4.41
		Retentate cooling	MJ/h	-0.63	-1.27	-2.71	-2.68	-2.65	-2.63	-2.63	-2.60	-2.61	-2.59
		Permeate cooling	MJ/h	-2.69	-4.47	-5.81	-5.98	-6.14	-6.29	-6.42	-6.60	-6.72	-6.89
		Distillation	MJ/h	-1550.88	-1550.05	-1552.76	-1559.93	-1563.51	-1559.27	-706.67	-1569.95	-708.36	-709.37
			MJ/h	1907.63	1907.22	1910.33	1917.67	1921.39	1917.31	1064.70	1928.27	1066.68	1067.84
		Heating sum	MJ/h	1910.77	1912.67	1917.34	1924.72	1928.49	1924.48	1072.02	1935.72	1074.25	1075.51
		Cooling sum	MJ/h	-1584.44	-1586.11	-1591.77	-1599.24	-1603.08	-1599.11	-746.66	-1610.35	-748.90	-750.19
		Total	MJ/h	326.32	326.56	325.57	325.48	325.41	325.37	325.36	325.37	325.35	325.32
